# Watching the availability and use of rapid diagnostic tests (RDTs) and artemisinin-based combination therapy (ACT)

**DOI:** 10.1186/s12936-017-1821-0

**Published:** 2017-04-24

**Authors:** Richard W. Steketee, Thomas P. Eisele

**Affiliations:** 1 Malaria Control and Elimination Partnership in Africa (MACEPA), PATH, Seattle, WA USA; 2 Department of Tropical Medicine, Center for Applied Malaria Research and Evaluation, Tulane University School of Public Health and Tropical Medicine, New Orleans, LA USA

## Abstract

At the turn of this new century and after much debate, the malaria community reckoned with failing first line therapies and moved to a global recommendation for deployment of an artemisinin-based combination therapy (ACT) to treat infections due to *Plasmodium falciparum.* No one said it was going to be easy. This series in the Malaria Journal reports longitudinal snapshots of how the core pillar of malaria elimination of ensuring universal access to malaria diagnosis and treatment is faring—it is safe to say “not so well”. Core issues that must be addressed to ensure universal access to diagnosis and treatment, and achieve elimination, include lack of access to these essential services for those with malaria and the lack of a common effective service delivery approach to ensure high quality diagnosis and treatment, especially in the private sector which provides the bulk of malaria case management services in many settings. The barriers to universal access to high quality diagnosis and treatment for malaria will need to be addressed if malaria elimination is to remain a real possibility in the foreseeable future.

No one ever said that changing the primary drug for a global infectious disease would be easy. At the turn of this new century and after much debate, the malaria community reckoned with failing first line therapies (e.g., chloroquine and sulfadoxine–pyrimethamine) and moved to a global recommendation for deployment of an artemisinin-based combination therapy (ACT) to treat infections due to *Plasmodium falciparum* [1]. Initially, artemether–lumefantrine (AL) was the only available oral ACT and it became the standard—and to this day, AL is the dominant ACT in the marketplace. But there are now many other options, including, unfortunately, many products that are not quality-assured ACT (QAACT) and, in fact, many counterfeit products pretending to be ACT medicines—whose use is both daunting and grave in its consequences [2, 3].

As this change was unfolding, many countries were experiencing progress in malaria control with the combination of increasing coverage and use of insecticide-treated mosquito nets (ITNs; now long-lasting and referred to as LLINs), improved point of care diagnosis with rapid diagnostic tests (RDTs) and the highly effective ACT. By 2007, the notion of malaria elimination and ultimate eradication was raised [4] and has gained momentum under the Global Malaria Technical Strategy [5]. A core pillar of this strategy is “Ensure Universal Access to Malaria Prevention, Diagnosis and Treatment” and one of the two supporting elements is “Strengthening the Enabling Environment” which includes the supply and logistics systems to provide access to necessary products, such as RDTs and ACT medicines.

In this Thematic Series of the Malaria Journal, authors provide longitudinal snapshots of how this pillar and supporting element are faring for the core requirements of diagnosis and treatment for malaria elimination—it is safe to say “not so well”. To their great credit, since its inception in 2008, the leaders of the ACTwatch programme have taken a rigorous, standardized, and population-based methodology [6] to a total of 12 countries in the Greater Mekong sub-region (GMS; n = 4) and sub-Saharan Africa (SSA; n = 8) in order to assess the availability of RDTs and/or ACT in these diverse settings (e.g., urban and rural, and public and private sector venues). This provides great confidence in the findings, increases generalizability, limits study bias, and allows the comparisons over time and across sites to remain robust. They have also provided summary reviews of the work in sub-Saharan Africa [7] and the Greater Mekong sub-Region [8] that nicely complement the mostly individual-country reports in this series. Of particular note, the work described in these papers is not easy. Clearly, many people in the different countries who have gone to great lengths and remote communities to obtain credible data that provides a wake-up call on the quality of diagnosis and treatment.

Many who are responsible for the access, delivery, and use of malaria testing and treatment will find this series of papers and other ACTwatch reports extremely useful in making critical decisions on improving malaria case management and the use of diagnosis and treatment in active case investigation. Below, some key issues raised in this series of papers are highlighted in the hopes of encouraging improvement of malaria case management in the coming years.

First, the progress is palpable—but incomplete. A decade after the introduction of ACT, there is significant improvement in the availability, affordability, quality and use of the ACT, especially in the public health service sector. Yet, as noted in a recent publication by Bennett and colleagues, while ACT coverage in children less than 5 years old with fever and *P. falciparum* infection increased during the 2003–2015 interval, treatment of those in need reached only 20% by 2015 [9]. Progress in access to ACT is heartening, but given the goal of eliminating malaria, this level of ACT coverage among those with malaria simply will not suffice.

Second, as the ACTwatch series shows, there is tremendous variation across countries, even neighbours, with the role of the private sector and the availability of parasitological diagnosis and ACT in the health system. This lack of consistency in policy and practice is indicative of the fact that we do not have a common effective service delivery approach to a core element of malaria control and elimination—the case management component and its extension into the community and the private sector. This core case management delivery strategy will also be critical as countries seek malaria elimination using additional community outreach, case investigation, and reactive case detection procedures to reduce infections and transmission.

Third, the series shows that in many countries, especially in urban areas, private sector providers are the source of the majority of malaria treatments. However, the private sector assessed in the ACTwatch studies too often lacked parasitological diagnosis and the exclusive use of QAACT, which is troubling to any control or elimination setting. In the context of improving quality of health service delivery for malaria, this misalignment creates a huge dilemma and challenge. In recent years, the malaria and public health community has generally recognized the importance of the private sector in making product available. Yet there has been limited investment and little capacity built to also address quality services in the private sector to achieve the goals of “test-treat-and-track” [10]—provide quality diagnostic testing to confirm suspected cases and only then provide QAACT. Of note, in the context of seeking malaria elimination, the private sector has historically never engaged in case and foci investigations to stop local transmission, so emphasis should be placed on quality case diagnosis and treatment in the private sector and then determine optimal private-to-public sector communication and collaboration to assure the additional required follow-up of cases. This series documents the major role of the private sector in providing commodities, but we must change the dynamic to achieve quality “care” and not just “product”.

The public sector, when it does function appropriately, appears to support the core of the quality that will be required for the future—both for control and reducing morbidity and mortality, and ultimately for the work required for malaria elimination. Country programmes and the wider malaria community have spent substantial time and energy getting National Malaria Strategic Plans, Global Fund Concept Notes, and US-President’s Malaria Initiative Malaria Operational Plans to systematically address quality case management as a core element of the work. Yet, these programmes (and the donor supporters) seem all too willing to accept the questionable quality in the private sector, as well as their inability to regulate or control the products and services in this sector. In the original WHO-led Malaria Eradication Programme of the 1950s and 1960s, they noted clearly that when countries reached pre-elimination and the “elimination end-game”, they should withdraw all anti-malarial drugs from the private sector so that the elimination programme could manage and control all diagnosis, treatment, reporting and case investigations. One can see why that was done; but in today’s world where value is placed on the balance and collaboration between public and private sector, further efforts are required to assure quality diagnosis and treatment of cases in both sectors as well as specifically supporting the public sector responsibility for complete case reporting and further case investigation and transmission containment.

It is notable that the original decisions that led to the introduction of a new first-line approach for malaria treatment—using ACT—established an evolving concern regarding malaria parasite resistance to available drugs. As a consequence, we have a global plan to combat anti-malarial drug resistance from the WHO and Roll Back Malaria Partnership [11], with regular updates [12], and a world-wide antimalarial resistance network (WWARN): http://www.wwarn.org/about-us/malaria-drug-resistance. Additionally, the critical multi-donor-supported Medicines for Malaria Venture is tasked with developing new drugs attending to target product profiles that anticipate their role and use in the future, including the means of reducing the risk of developing resistance.

The malaria community’s efforts to fend off anti-malarial drug resistance is laudable and will be required for the future as resistance will certainly evolve. However, the data presented in this series should make us recognize that a commensurate investment is required to systematically assure quality diagnostics and quality drugs are available and accessible wherever malaria is transmitted. It may be time to develop “target delivery profiles” that bring the same rigour to the delivery systems that are demanded from the diagnostics and drug research and development community. Such attention to quality and rigour will likely save additional lives and reduce infections and transmission at the same time that it will actually help with the future durability of drug efficacy.
